# Shared decision making in consultations for hypertension: Qualitative study in general practice

**DOI:** 10.1111/hex.13234

**Published:** 2021-04-05

**Authors:** Rachel Johnson, Katrina Turner, Gene Feder, Helen Cramer

**Affiliations:** ^1^ Centre for Academic Primary Care, Population Health Sciences Bristol Medical School Bristol UK

**Keywords:** decision making, hypertension, primary health care, qualitative research, shared

## Abstract

**Background:**

Hypertension is mainly managed in primary care. Shared decision making is widely recommended as an approach to treatment decision making. However, no studies have investigated; in detail, what happens during primary care consultations for hypertension.

**Aim:**

To understand patients’ and clinicians’ experience of shared decision making for hypertension in primary care, in order to propose how it might be better supported.

**Design:**

Longitudinal qualitative study.

**Setting:**

Five general practices in south‐west England.

**Method:**

Interviews with a purposive sample of patients with hypertension, and with the health‐care practitioners they consulted, along with observations of clinical consultations, for up to 6 appointments. Interviews and consultations were audio‐recorded and observational field notes taken. Data were analysed thematically.

**Results:**

Forty‐six interviews and 18 consultations were observed, with 11 patients and nine health‐care practitioners (five GPs, one pharmacist and three nurses). Little shared decision making was described by participants or observed. Often patients’ understanding of their hypertension was limited, and they were not aware there were treatment choices. Consultations provided few opportunities for patients and clinicians to reach a shared understanding of their treatment choices. Opportunities for patients to engage in choices were limited by structured consultations and the distribution of decisions across consultations.

**Conclusion:**

For shared decision making to be better supported, consultations need to provide opportunities for patients to learn about their condition, to understand that there are treatment choices, and to discuss these choices with clinicians.

**Patient or Public Contribution:**

A patient group contributed to the design of this study.

## 1 INTRODUCTION

Shared decision making is a collaborative process through which a clinician supports a patient to reach decisions about their health‐care treatments.^1^ There are many models of shared decision making;^2^, ^3^, ^4^, ^5^ a recent influential model describes a process in which information is exchanged between clinician and patient about treatment options, and the patient's values and preferences, before choices are deliberated in collaboration.^5^

The NHS long‐term plan aspires to make shared decision making the usual experience for patients.^6^ However, shared decision making has proven challenging to embed in routine care;^7^, ^8^, ^9^ clinicians report time constraints and shared decision making not applying to all clinical situations.^10^ Although providing information about treatment options can increase knowledge about those options,^11^ this does not necessarily enable patients to participate in discussions or decisions about their care, and patients report feeling disempowered within consultations.^9^ Challenges for shared decision making in UK primary care include that consultations often cover several problems,^12^ diagnoses may be unclear, and the consultation may not focus on choosing appropriate biomedical treatment.^13^ The distribution of decisions over time, courses of action, people and situations ^14^, ^15^, ^16^ might also present challenges for shared decision making. While some UK initiatives have shown promise in facilitating shared decision making,^8^ few studies have focused on shared decision making for hypertension in the primary care setting.

Hypertension increases the risk of cardiovascular conditions such as strokes and heart attacks and is the leading preventable cause of premature death worldwide.^17^ In the UK, hypertension affects 14% of adults^18^ and is managed mostly in primary care, accounting for 12% of primary care consultations and approximately £1 billion in drug costs in 2006.^19^ Recommended management options are based on age, blood pressure level and absolute risk of cardiovascular events and include drug treatments (anti‐hypertensives and statins) and lifestyle modification.^20^ Treatment is typically lifelong and adjusted over time. Optimal blood pressure targets vary internationally and by comorbidity and are the subject of vigorous debate.^21^ Hypertension control has long been considered suboptimal; that is, it fails to reach specified treatment targets.^22^, ^23^

Blood pressure lowering reduces cardiovascular risk and delivers cost savings at the population level.^24^, ^25^ The likelihood of benefit / dis‐benefit varies with cardiovascular risk and with the threshold at which hypertension is diagnosed and treatment considered. Shared decision making has the potential to ensure decisions are based on what matters to patients, informed by their assessment of the potential benefits and harms.

This study aimed to explore primary care patients’ and clinicians’ experiences of decision making for hypertension treatment, in order to understand if and how patient involvement in decision making might be supported.

## 2 METHODS

We chose a longitudinal design and qualitative methods to facilitate in‐depth exploration of patients’ and clinicians’ experiences of decision making. Our aim was to follow patients as they attended health‐care consultations and to understand the consultations both from their perspective and from the perspective of the clinician they had consulted. We did this using baseline interviews with patients at entry to the study, observations of consultations with a range of health‐care practitioners and post‐consultation interviews with patients and clinicians.

### 2.1 Sampling and recruitment

We used purposeful sampling^26^ to recruit practices varying in socio‐demographic characteristics and organizational structures in a large southern English city. Within practices, we purposively sampled patients 18 years or older with hypertension to achieve a sample with a range of ages and at different stages after a diagnosis of hypertension. Adults with hypertension were identified using electronic record searches, mailed study information and asked to contact the research team if interested in the study. Sequential recruitment of patients and practices allowed sampling to be informed by early findings. All patients and health‐care professionals provided written informed consent. The concept of ‘information power*’* [24] was used to inform the patient sample size required. This meant data collection ended when the sample held sufficient information relevant to the study, to address its aims.

### 2.2 Data collection

Data were collected between May 2017 and March 2018. All data were collected by RJ, a female clinician (general practitioner) and researcher, who was not involved at any stage in the clinical care of the patient participants or as a practitioner at the practices involved. One of the practitioners (GP, observed in 1 consultation) was known to RJ on a professional level. All included patients took part in a baseline interview. Patients informed the researcher about their health‐care appointments, so that they could be observed and audio‐recorded. Following consultations, health‐care professionals and patients were interviewed separately to understand their perspectives on the consultation. Brief topic guides were used for both baseline and post‐consultation interviews. Patient participants were followed for up to six health‐care appointments to allow observation of experiences over time. Field notes recorded observations about the health‐care setting and consultations and served as a prompt for post‐consultation interviews. Baseline and post‐consultation interviews were audio‐recorded, and, with a small number of exceptions (due to researcher unavailability or technical failure of recording equipment), consultations were both observed and audio‐recorded.

### 2.3 Data management and analysis

Interview and consultation data were audio‐recorded, transcribed verbatim and anonymized. NVivo was used to aid data management and analysis. Data analysis was thematic,^27^ used an inductive, constant comparison method and was concurrent with data collection. Three authors (RJ, HC and KT) developed and refined the coding frame. KT and HC are qualitative methodologists: KT has a disciplinary background in social sciences applied to health; HC has a background in social and medical anthropology.

Data for each participant were analysed as a case. Data were explored for similarities and differences within patient case studies (between patients and clinicians, between consultations and post‐consultation interviews), over time, and between case studies. Codes were built into broader categories and themes; themes were developed inductively from analysis across the case studies.

As data collection continued, we re‐assessed the factors influencing information power^28^ and ended data collection when we judged sufficient information power had been achieved.

We chose not to use a specific theory of shared decision making to inform the analysis, as we were keen for our findings to be inductively developed from the data. Also, a systematic review of shared decision‐making models found little agreement on the concept, identifying only two features (patients values / preferences, and options) appearing in more than half of the models.^2^ We therefore adopted a broad understanding of shared decision making, that is decision making in which both patients and health‐care professionals are involved, while sensitizing our analysis to the presentation of options and discussion of patients’ values and preferences.

The study Patient and Public Involvement group comprising seven members (four patients with cardiovascular disease and three of their partners / carers) assisted in development of the study materials and in the submission of an ethics application, which was reviewed and approved by the NHS ethics committee (Research Ethics Committee reference: 16/SW/0294REF).

## 3 RESULTS

### 3.1 Participants

Five general practices in Bristol, England, were recruited. Eleven patients with hypertension were recruited; participant characteristics and data collection are summarized in Table 1. All participants were white. Patients’ and GP’s names have been replaced with pseudonyms to maintain confidentiality. Eighteen consultations were audio‐recorded/observed (3 with practice nurses, 3 with clinical pharmacists and the remainder with GPs), and 35 post‐consultation interviews (20 with patients and 15 with health‐care professionals were carried out.

**TABLE 1:**
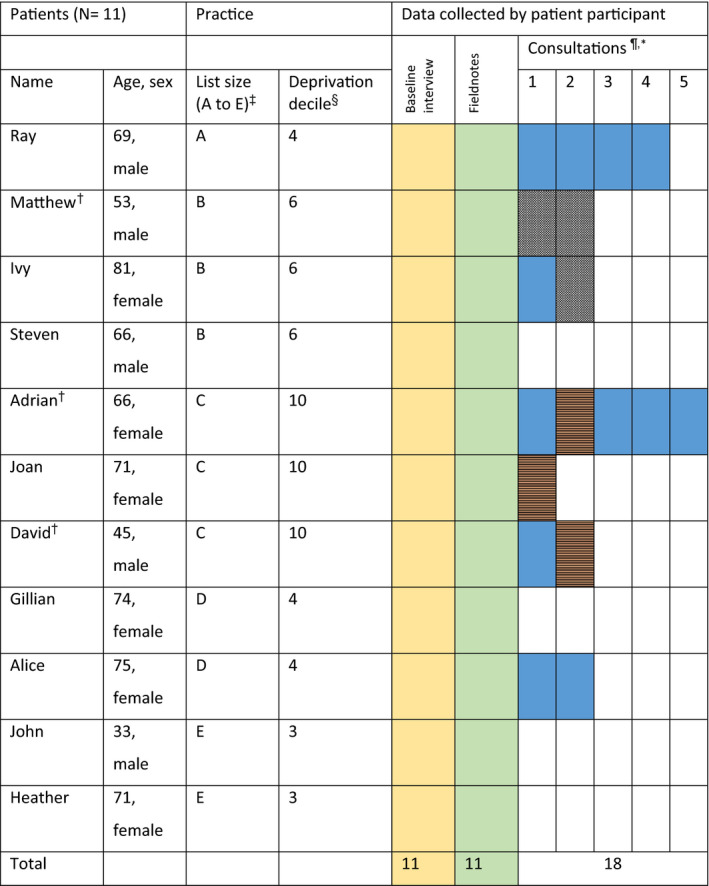
Overview of practices, patient participants and data collected

^*^Health‐care practitioner key: General Practitioner 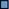; Practice nurse 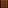; No consultation 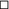.

The yellow and Green boxes just indicated that all patients had those items of data collection. Black and white hashed box indicates a consultation with a clinical pharmacist.

^†^Indicates diagnosis within the last year. NB Pseudonyms are used for all participants.

^‡^Practice list size: A = 7979, B = 8794, C = 9732, D = 15 359, E = 11 312.

^§^Lower number = more deprived.

^¶^Three consultations were not recorded because of practical issues (researcher not available).

### 3.2 Results of thematic analysis

**FIGURE 1:**
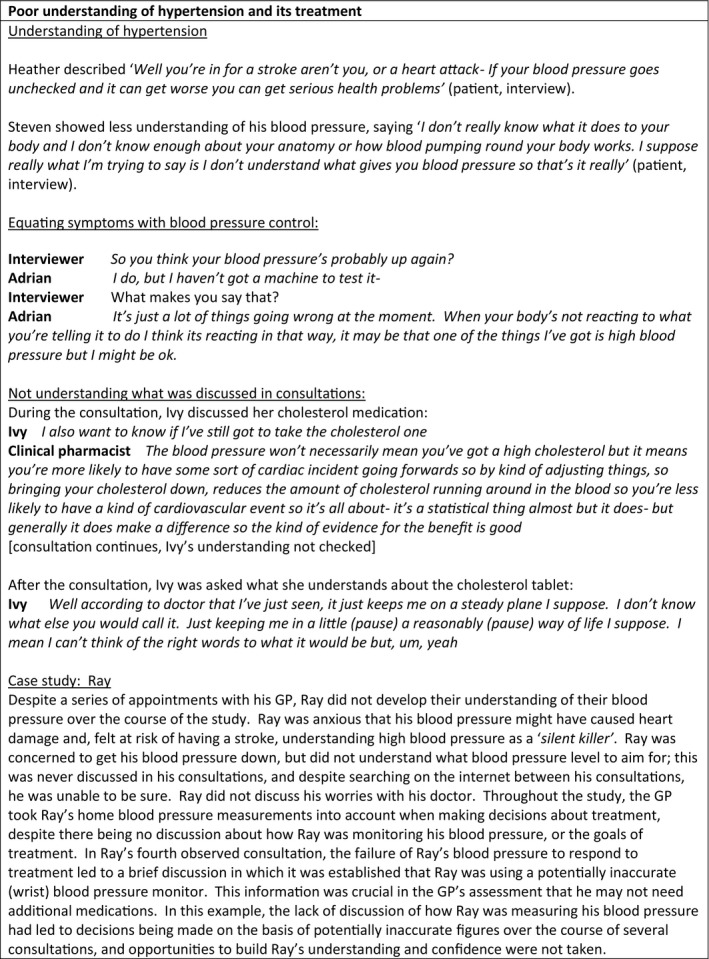
Poor understanding of heart failure and its treatment

**FIGURE 2:**
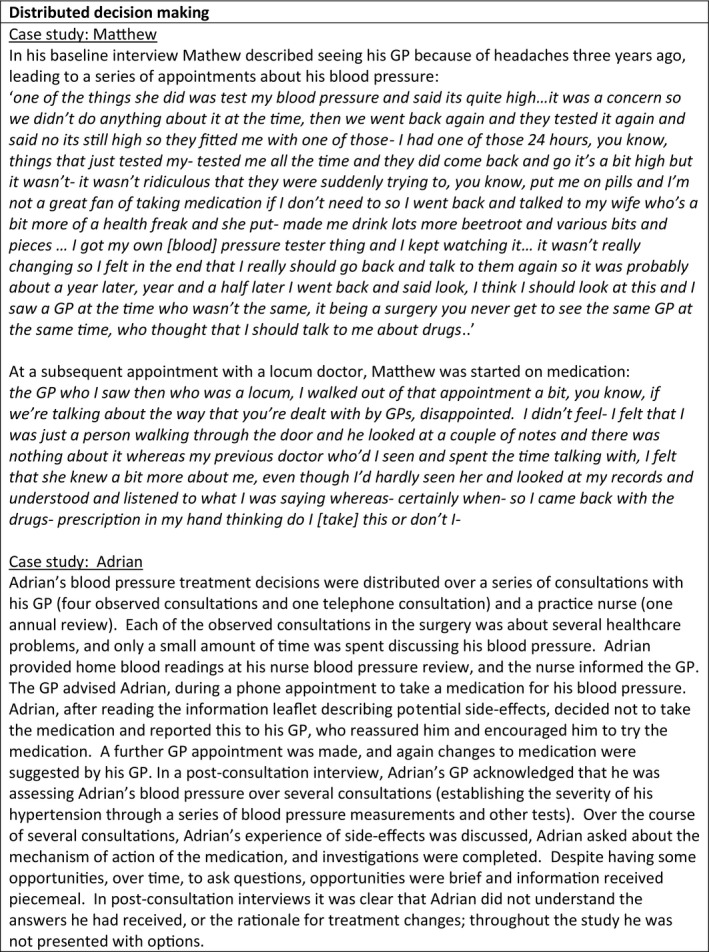
Distributed decisions

**FIGURE 3:**
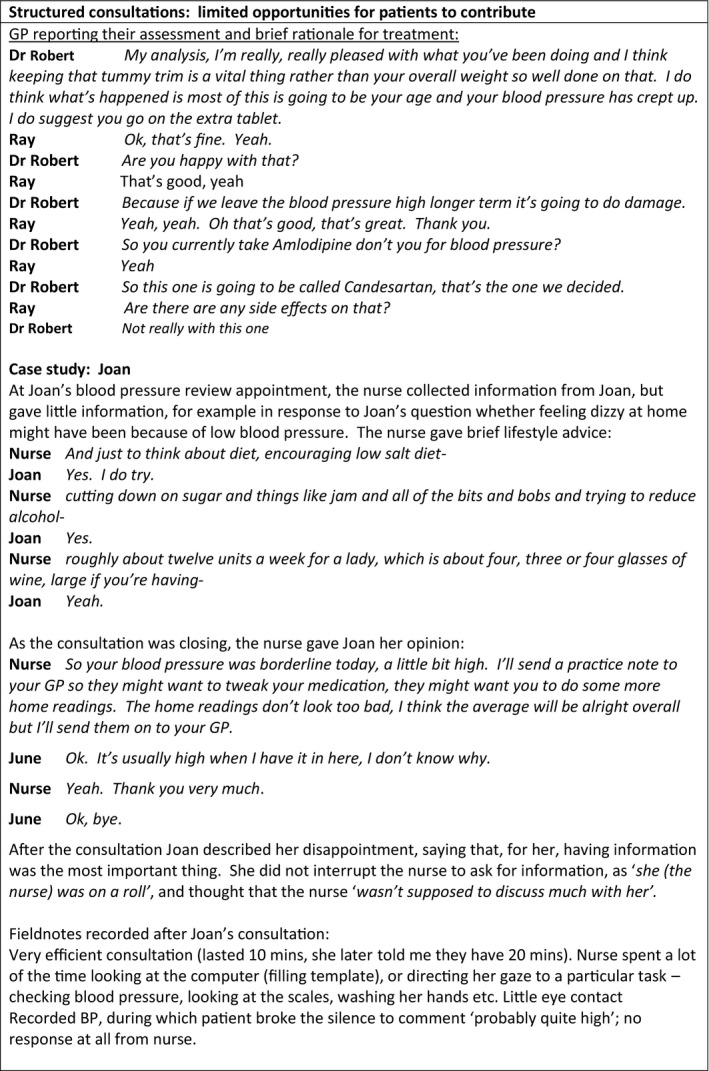
The constraints of structured consultations

**FIGURE 4:**
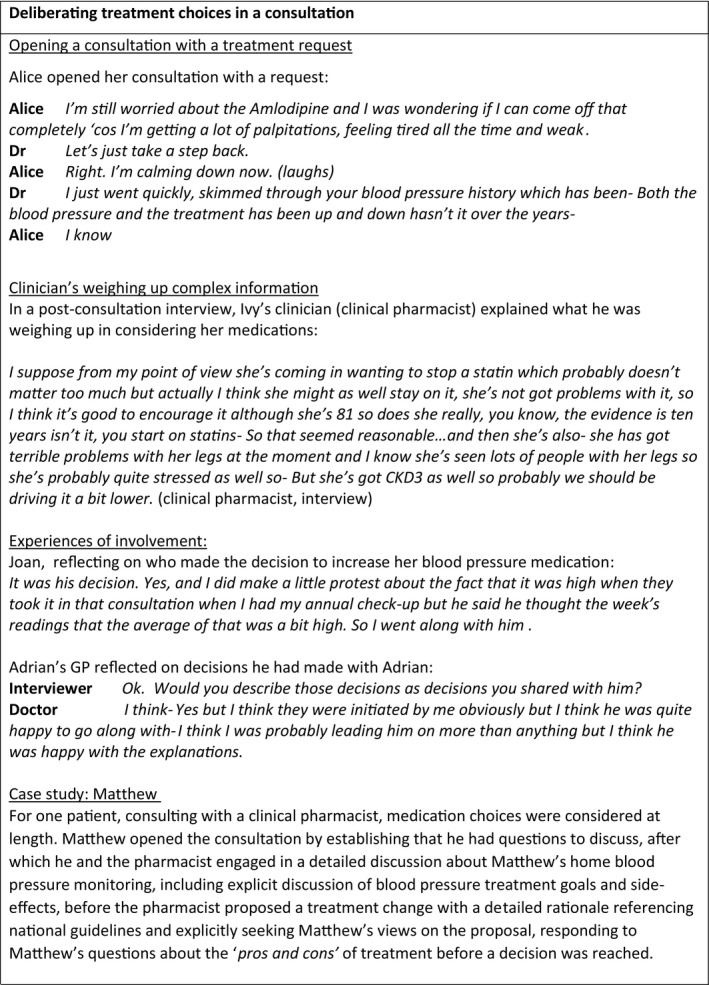
Limited involvement in deliberating treatment choices

In both interviews and consultations, decisions reported in relation to hypertension treatment were starting, stopping and increasing the dose of anti‐hypertensive medications; starting statin therapy; and decisions about diet and exercise. Findings are reported in four themes: poor understanding of hypertension and its treatment, distributed decisions, perceived lack of choice, and the limited opportunities for patient involvement in decision making. Illustrative quotes, tagged with information about the interviewee (patient/clinician) and method of data collection (interview/observation) are provided in the main text, with additional quotes, consultation examples and case studies in Figures 1, 2, 3, 4.

### 3.3 Poor understanding of hypertension and its treatment

During the interviews, patients reported understanding that having high blood pressure meant they might have a heart attack or stroke **(**Figure 1 **)**. However, understanding of high blood pressure and cardiovascular risk was often limited. Patients reported lack of time and discomfort at asking questions as reasons they did not ask questions in consultations. Alice described ‘*no, you haven't always got the chance to speak to somebody that understands the tablets you're on…you're only given a certain amount of time when you go and see a doctor and their time's took up*’ (patient, interview). Heather explained: ‘*to be honest they didn't discuss it with me to say why they were using, what the tablets were for…because I used to think ‘what does this one do’ and ‘what would happen if I start taking it’ but I never had that rapport to tell me…I just couldn't talk to him…he just had a very offhand way’* (patient, interview).

Some patients attributed symptoms to their high blood pressure and used symptoms to work out if their blood pressure was sufficiently treated, while others thought that hypertension treatment was temporary and expected that it would be stopped. Most patients did not know what their blood pressure or blood pressure goal was. One patient (Matthew) stood out as having good understanding of his hypertension and his blood pressure goals, an understanding which he attributed to accessing information outside health‐care appointments. While several patients measured their blood pressure at home, only Mathew was clear what his blood pressure should be, and some did not discuss their home monitoring in consultations. Adrian explained that he had sought information online: *‘they didn't ask me ‘do you know about high blood pressure’, that was never asked, it's like you have high blood pressure, go away and get it lower. This is what you do. Yeah, there is no‐ well I certainly didn't have any information, that's it. I think they assumed that you know it basically’*. Asked if he knew how to interpret his blood pressure values, he answered *‘Yes, don't understand them at all’* (patient, interview).

Most observed consultations provided little opportunity for patients to ask questions, discuss or develop their understanding of hypertension and its management, despite some having multiple consultations, sometimes with a range of providers. In none of the consultations observed was the patient's understanding sought or checked by the health‐care practitioner. Often, during consultations and post‐consultation interviews, it was evident that patients had not understood what had been discussed, that patients’ and clinicians’ understandings of hypertension and its treatment were different and that both had made assumptions about the understanding of the other that were not explored in consultations. For example, blood pressure was often measured in consultations but the result, and the goals of treatment, was rarely discussed. After a consultation in which Ray's blood pressure had been measured, Ray described how he tried to judge whether his blood pressure was controlled by observing his GP’s reaction: ‘*you can tell a lot by people's face…looking at [GP] to see if his eyes went open if the blood pressure was really bad*…*I think it was good, cos there was nothing in his face to show that there was a, you know, it was high or whatever’* (patient, interview).

### 3.4 Distributed decisions

Decisions about treatment were often distributed, that is, as an on‐going event evolving across encounters and over a range of people (Figure 2).^15^ They were also sometimes revisited, over time and between different consultations and different health‐care professionals. Most decisions were made by GPs, but the processes of decision making, notably collecting information relevant to a decision, involved other health‐care professionals, for example nurses. Sometimes information relevant to a decision was collected on one occasion, but informed a treatment discussion on another occasion. Treatments might be suggested in one consultation, and decisions made in subsequent consultations, including sometimes over the telephone, and sometimes by different practitioners. In some instances, patients or clinicians made decisions about whether to begin or stop treatment between consultations. For example, David stopped his statin medication, perceiving side‐effects: ‘*I was getting tingling sensations in my fingers and I was dizzy and I thought oh the only thing I’m taking is the statins so I stopped taking the statin*’ (patient, interview). Some GPs were observed to test the patient's response to a treatment suggestion, and if the patient seemed reluctant, to leave the discussion for a subsequent consultation. Ivy said that she wanted to reduce her blood pressure medication, opening the consultation by saying ‘*now I was actually hoping this time that instead of the two lots of blood pressure tablets I could change it to just one’* (patient, observation). The clinician wanted to increase her medication and tried to prepare Ivy for this in future, reflecting afterwards ‘*with the Lisinopril [blood pressure tablet], wanting to drop that was clearly not the place to go was it ‘cos she was on‐ her BP’s still high so actually I’d rather‐ I was tempted to move it up but I thought when she came in going ‘well I want to drop one of my Lisinoprils’ you're thinking oh, she's not going to be that amenable to increasing so‐ So I think probably like give her some time to think about it, hopefully I’ve sowed the seed. That was kind of my thought process*’ (clinician, interview).

Information about side‐effects was also sometimes given over a series of consultations, and the effects of treatment changes were reviewed over time. The different competencies of practitioners that patients consulted sometimes led to patients receiving confusing information. David described frustration during a consultation with a practitioner who he felt was not appropriately qualified: ‘*she was looking at them [his blood test results] and …she said oh that looks a bit pre‐diabetic to me. I was like [swears] ok, so tell me what's that about. She said well ok, talk to your GP about that. I was like no [laugh] I want to talk about it right now if you're going to say that to me’* (patient, interview). Distributed decision making appeared to obscure the decisions being made and exclude the patient from involvement in decision making.

### 3.5 Perceived lack of choice

Most patients reported that clinicians made the decisions. Most patients understood treatment changes to be necessary, appeared to accept them with little discussion and did not perceive that there was a choice to be made. Trust in doctors’ expertise helped some to accept treatment, for example John said ‘*You got to ask questions to see what you got to take the tablets for or not take the tablets for, but the doctor's always right’* (patient, interview). When asked about whether there was a choice about her anti‐hypertensive treatment, Joan responded ‘*No, because I went along with whatever the doctor said. I wouldn't dream of refusing to take them…I think the doctor knows best, you hope’* (patient, interview). Only one patient (Matthew) recalled the decision to start an anti‐hypertensive as one he could make, allowing him to choose to focus on lifestyle changes rather than start medication. Matthew said: ‘*she [the GP] wasn't sitting there going you've got to do this or you're going to die, it was like well look we need to keep an eye on it but you know, if you don't want me to add anything or prescribe you anything then I won't’* (patient, interview).

Patients described two circumstances in which they took a less passive approach. Firstly, experiencing side‐effects often prompted patients to question a medicine's suitability. Secondly, when patients were aware of treatment alternatives they were more likely to engage in a discussion about treatment. This was most evident in relation to decisions about cholesterol‐lowering (statin) therapy: several patients had views on statins, informed by press reports and discussions with friends and family. For Ray (interview), statins were a ‘*wonder drug’* that he was keen to start, while David (interview) had decided that statins ‘*do more harm than good’* and he did not want to take them, and for Matthew (interview) ‘*statins was a word I knew and understood and therefore typed in before I went to see the doctor for the first time’*. This awareness sometimes led to discussions about treatment, as captured by Gillian (interview): ‘*I know there's a great argument about cholesterol…we did have quite a discussion about that’*. However, being aware of these uncertainties did not necessarily mean patients had helpful discussions with their clinicians. Ray was disappointed when given a prescription for statins as he had not had time to discuss in detail their pros and cons, while Gillian, despite having a conversation with her doctor, found it difficult to make a choice, saying ‘*there are pros and cons and that puts you in a difficult position as a patient…I had to rely ultimately on what the doctor said…I asked him ‘would you take it in my shoes?’ and he said ‘yes’, I thought well what do I do? And I thought yes, I will’* (patient, interview).

### 3.6 The limited opportunities for patient involvement in decision making

#### 3.6.1 The constraints of structured consultations

Consultations with a range of health‐care practitioners were observed to be highly structured and led by the health‐care practitioner (Figure 3). The consultation structure typically began with a greeting, after which the practitioner asked a series of questions to collect information, followed by a physical assessment. Observed consultations with nurses included routine yearly reviews. During these, data collection focused on lifestyle behaviours and examination included measurements such as blood pressure and weight. Asked after a consultation what her role was, one nurse responded ‘*just to literally – go through the template, just ask them about the diet and exercise, just to inform them what I’m doing’* (nurse, interview). Brief lifestyle advice was given in response to behaviours reported, and deviations from the routine were infrequent and brief. In consultations with GPs, information gathering included symptoms, medication taking, side‐effects and lifestyle behaviours. Physical assessment was often followed by the GP reviewing medication lists and recent investigations, before reporting their findings and making a plan.

Consultation routines were practitioner‐led, with patients providing responses to the practitioner's questions. Routines appeared to constrain the opportunities for the patient to contribute to the consultation. As Ivy commented, ‘*consultations are about one specific thing and the doctor pulls you back to* these’ (patient, interview). At the beginning of the consultation, some clinicians opened the consultation with a question asking the patient what they wanted to address. David's GP opened the consultation by asking ‘*what do we want to start with?*’(clinician, observation). Typically, later questions to the patient were seeking agreement to the clinician's suggestions, or checking that the patient had no further questions. Patients reported that the structured nature of the consultation was frustrating.

#### 3.6.2 Limited involvement in deliberating treatment choices

During observed consultations, changes to treatment were usually suggested by health‐care professionals (pharmacists and doctors) after they had completed their assessment (Figure 4). Treatment proposals were sometimes preceded by a brief summary of the assessment and rationale for the suggested change. Occasionally patients opened the consultation with a proposed treatment change, sometimes triggered by the experience of side‐effects; when they did so, this often led to a change in medication.

Following a treatment proposal by either patient or clinician, only a very small amount of consultation time was devoted to discussing the proposal and making a decision about a treatment. Clinicians typically suggested one treatment without alternatives, or occasionally more than one option (eg to increase medication or pursue lifestyle changes). When GPs wanted to increase a patient's hypertension medication, in no instances did they offer the option of not increasing it, or offer alternative anti‐hypertensive medications. Reflecting on a decision to increase her blood pressure medication Joan said : ‘*He didn't suggest anything else’*, and when asked if side‐effects were discussed she answered ‘*No. Didn't think about that’* (patient, interview). There was no detailed discussion of risks and benefits of treatments, and in only one observed consultation was the patient asked for their perspective on what they wanted to achieve, their treatment preferences, the proposed treatment or alternative treatment options. Observations and post‐consultation interviews with clinicians indicated that, often, many decisions were being deliberated by clinicians as they assessed the patient, but these deliberations were not made explicit or shared with patients.

During the interviews, many patients reported satisfaction with their involvement in decision making. John said ‘*you want the best decision for yourself really, that's why‐ she tells you to take they tablets, you're putting your hand in hers to try and sort it out ‘cos she's a doctor’* (patient, interview). For some, agreeing to a treatment or participating in a brief discussion about treatment was perceived as involvement in the decision. However, some patients reported being dissatisfied with decision making in consultations. For example, Adrian expressed frustration at being only offered increasing amounts of one medication, over a series of consultations, to lower his blood pressure, saying ‘*You never really have enough time to ask other questions and get another solution. Is there any other thing I could take apart from Amlodipine, he's already chosen the best for me I suppose and you've got to trust the doctor’*. Alice, who had developed new health problems attributed to a blood pressure medication that she had insisted remaining on, was angry that her GP had appeared to accept her request without discussion. Neither Adrian nor Alice discussed this with their GP.

Many doctors felt that patients would be able to voice their disagreement with the doctor, if needed. When side‐effects were experienced, some GPs reflected that the right course of action (to change the treatment) was evident, while for treatments that aimed to prevent future events rather than manage a symptom, GPs were more likely to lead the decision. One clinician described how some choices were for the patient to make (whether to persist with lifestyle choices or accept increase anti‐hypertensive medication), but if blood pressure was persistently above target after this, the decision making became the realm of the clinician: ‘*In truth the majority of this now is my decision making based on his average blood pressure reading and it's a matter of getting him on board and understanding the need to take the treatment…so it's just sharing the data and helping him to understand the need to get better control as it is poorly controlled…in truth the hard decisions are mine but I would like him to be‐ to feel that he understands the reasoning behind my decisions’ Dr Robert, Ray's GP*.

## 4 DISCUSSION

### 4.1 Summary

Little shared decision making was described by patients or observed in consultations. Often, patients’ understanding of their hypertension was limited and they were not aware that choices about treatment (including the option of no treatment) existed. Consultations provided few opportunities for patients to develop their understanding and, with their clinician, to reach a shared understanding of their treatment choices. Opportunities for patients to engage in choices were limited by the structured nature of most consultations which constrained most patients’ contributions to responses to information requests, and the distribution of decisions across consultations. Choices about statins were an exception as several patients understood this as a choice. Clinicians were understood, by both patients and clinicians, to be the main decision makers.

### 4.2 Strengths and limitations

The use of multiple qualitative methods and a longitudinal design allowed us to explore decision making from multiple perspectives, to understand how, when and why decision making did or did not occur, and to do so over time. It is accepted that the presence of a clinically trained observer in consultations might influence the behaviour of practitioners and patients.^29^ Participants were aware that the study focused on choices in consultations. This may have shifted clinician behaviour either towards more involvement in choices, or towards increasing treatment (ie away from shared decision making); despite this, little shared decision making was observed. Not all patients had consultations during the study, and logistical problems made it impossible to audio‐record a small number of consultations. Although the dataset as a whole is rich, comprising 11 in‐depth interviews, 16 observed consultations and 35 post‐consultations, the number of consultations makes it difficult to draw conclusions, for example, about the differences between different health‐care professionals. All participants were white, despite attempts to recruit from more ethnically diverse practices.

The themes in this study were developed in relation to patients with hypertension, although consultations were often complex and many different problems were discussed. This focus allowed exploration of how the nature of the health condition (hypertension) impacted on involvement in shared decision making. However, the themes are likely to be generalizable to wider experiences of shared decision making in primary care, for example decisions about anticoagulation therapy in atrial fibrillation, or choice of anti‐hyperglycaemic treatment in type 2 diabetes.

### 4.3 Comparison with existing literature

Few studies in primary care have used observational methods to explore shared decision making and we are not aware of any focusing on hypertension decision making. While shared decision making is endorsed widely in national guidance,^30^, ^31^ robust evidence to guide a choice of intervention to support shared decision making for hypertension, or to understand is health outcome, is lacking.^32^ In this study, as in others, little shared decision making was observed,^33^ yet some patients were satisfied with their limited role in treatment decisions. This may reflect a true preference not to be involved. Alternately, it may reflect an internalized set of normative expectations about their participation,^34^ or the desire to be a ‘good patient’ and avoid conflict within the encounter.^9^ Elwyn et al’s collaborative deliberation model^5^ describes how, in consultations, exchanging information about treatment options, values and preferences facilitate collaborative deliberation about choices between clinician and patient. In this study, information exchange about options, values and preferences was minimal, and deliberation about choices was rarely observed. Qualitative studies in different contexts report that patients prefer collaboration, including sharing concerns and receiving explanations, but many feel they lack sufficient expertise, or power, to make decisions,^9^, ^35^ and often clinicians do not explicitly mention or provide detailed information about treatment options.^36^, ^37^ In this study, clinicians expected that patients would feel able to voice any concerns about their medicines. However, a recent study found that affluent primary care patients feared being dismissed or labelled as difficult if they challenged clinicians’ authority or expertise and therefore could not rely on physicians to help them understand treatment options.^38^

Decisions about high blood pressure treatment are often guideline‐based and in this study were viewed as straightforward transactions and often proceeded without discussion of uncertainty about the best course of action, or explicit acknowledgement that there was a choice. A recent qualitative study highlighted that older participants vary widely in their health goals and preferences for treatment outcomes from treatment of cardiovascular conditions.^39^ Acknowledging options explicitly may support patients to consider their values and preferences. Recent NICE guidance ^40^ (published after data collection for this study) includes decision aids for choice of first anti‐hypertensive medication; in this study, no decision aids were used. Survey data suggest that primary care clinicians’ knowledge of the benefits and harms of common long‐term condition treatment is poor, with inaccuracies of a magnitude likely to meaningfully affect clinical decision making and affect conversations with patients;^41^ this needs to be addressed to facilitate sharing of information about treatment options.

Clinicians follow a structured approach to consultations, formalized in consultation models, one aim of which is to assist the clinician in negotiating the complexity of the interaction between patients and doctors.^42^ Prominent models establish the consultation as task‐focused, while encouraging patient‐centred elements such as exploring the patient's ideas, concerns and expectations.^43^, ^44^ In this study, consultation routines could be discerned and appeared to be used to manage consultations efficiently by ensuring that biomedical tasks were addressed, but were infrequently used to focus on probing patient concerns. Opening up space in consultations to better understand patient perspectives on treatment may be necessary for shared decision making, but may have consequences for the efficiency of consultations to achieve other important tasks. In this study, we described distributed decision making ^14^, ^15^, ^16^ which made it more difficult to identify decisions and resulted in a lack of transparency and the exclusion of patients from the decision‐making space.^16^

### 4.4 Implications for research and clinical practice

The provision of universal personalized care, including shared decision making, is a central tenet of the NHS 10‐year plan.^6^ For shared decision making to happen, treatment options must be identified and made explicit. More work is needed to clarify which decisions should be shared between doctor and patient, and how best to do this. Future work could analyse consultations for hypertension using validated shared decision‐making tools. As a minimum, patients should be aware when decisions are being made about them, and should understand how the information they share with clinicians is taken into account when decisions are made. For shared decision making to be supported, patients need better understanding of their hypertension, and their understanding and treatment preferences need to be explicitly sought. Shared decision making is not possible when patients and clinicians do not have a shared understanding of the decision they are facing and patients do not understand what is at stake when decisions are made.

Efforts to support shared decision making need to focus on how consultations are organized, and how decisions arise within, and across consultations. It is important to identify the potential ways in which shared decision making could be supported in the context of distributed decision making, which is likely to be commonly encountered in primary care. For example, distributed decision making might provide multiple opportunities for patients to develop informed preferences, taking into account perspectives from a range of health‐care providers, provided the decisions being considered are made explicit, and health‐care professionals have the required skills.^14^ Consultations need to achieve biomedical tasks alongside addressing the patient's agenda. Given the inherent power imbalance in the doctor–patient relationship,^9^ this may require re‐structuring consultations, in order to make patients more equal partners in the conversation.

## 5 ETHICS APPROVAL

This study was reviewed and approved by the NHS ethics committee (Research Ethics Committee reference: 16/SW/0294REF).

## CONFLICTS OF INTEREST

The authors declare no conflicts of interest.

## Data Availability

Data available on request due to privacy/ethical restrictions: The data that support the findings of this study are available on request from the corresponding author. The data are not publicly available due to privacy or ethical restrictions.

## References

[1] Shared decision making. NHS England. https://www.england.nhs.uk/shared‐decision‐making/. Accessed 20 September, 2019.

[2] Makoul G , Clayman ML . An integrative model of shared decision making in medical encounters. Patient Educ Couns. 2006;60(3):301‐312. 16051459 10.1016/j.pec.2005.06.010

[3] Charles C , Gafni A , Whelan T . Shared decision‐making in the medical encounter: what does it mean? (or it takes at least two to tango). Soc Sci Med (1982). 1997;44(5):681‐692. 10.1016/s0277-9536(96)00221-3 9032835

[4] Elwyn G , Durand MA , Song J , et al. A three‐talk model for shared decision making: multistage consultation process. BMJ. 2017;359;j4891. 29109079 10.1136/bmj.j4891 PMC5683042

[5] Elwyn G , Lloyd A , May C , et al. Collaborative deliberation: a model for patient care. Patient Educ Couns. 2014;97(2):158‐164. 25175366 10.1016/j.pec.2014.07.027

[6] The NHS Long Term Plan. In: NHS England; 2019.

[7] Elwyn G , Scholl I , Tietbohl C , et al. “Many miles to go …”: a systematic review of the implementation of patient decision support interventions into routine clinical practice. BMC Med Inform Decis Mak. 2013;13(S2):S14. 24625083 10.1186/1472-6947-13-S2-S14 PMC4044318

[8] Joseph‐Williams N , Lloyd A , Edwards A , et al. Implementing shared decision making in the NHS: lessons from the MAGIC programme. BMJ. 2017;357:j1744. 28420639 10.1136/bmj.j1744 PMC6284240

[9] Joseph‐Williams N , Elwyn G , Edwards A . Knowledge is not power for patients: A systematic review and thematic synthesis of patient‐reported barriers and facilitators to shared decision making. Patient Educ Couns. 2014;94(3):291‐309. 24305642 10.1016/j.pec.2013.10.031

[10] Légaré F , Ratté S , Gravel K , Graham ID . Barriers and facilitators to implementing shared decision‐making in clinical practice: Update of a systematic review of health professionals’ perceptions. Patient Educ Couns. 2008;73(3):526‐535. 18752915 10.1016/j.pec.2008.07.018

[11] Stacey D , Légaré F , Lewis K , et al. Decision aids for people facing health treatment or screening decisions. Cochrane Database Syst Rev. 2017;4(4):CD001431. 28402085 10.1002/14651858.CD001431.pub5 PMC6478132

[12] Salisbury C , Procter S , Stewart K , et al. The content of general practice consultations: cross‐sectional study based on video recordings. Br J Gen Pract. 2013;63(616):e751‐e759. 24267858 10.3399/bjgp13X674431 PMC3809428

[13] Murray E , Charles C , Gafni A . ,Shared decision‐making in primary care: Tailoring the Charles et al. model to fit the context of general practice. Patient Educ Couns. 2006;62(2):205‐211. 16139467 10.1016/j.pec.2005.07.003

[14] Joseph‐Williams N , Williams D , Wood F , et al. A descriptive model of shared decision making derived from routine implementation in clinical practice (‘Implement‐SDM’): Qualitative study. Patient Educ Couns. 2019;102(10):1774‐1785. 31351787 10.1016/j.pec.2019.07.016

[15] Rapley T . Distributed decision making: the anatomy of decisions‐in‐action. Sociol Health Illn. 2008;30(3):429‐444. 18194358 10.1111/j.1467-9566.2007.01064.x

[16] Ofstad EH , Frich JC , Schei E , Frankel RM , Gulbrandsen P . Temporal characteristics of decisions in hospital encounters: a threshold for shared decision making? A qualitative study. Patient Educ Couns. 2014;97(2):216‐222. 25176608 10.1016/j.pec.2014.08.005

[17] Forouzanfar MH , Alexander L , Anderson HR , et al. Global, regional, and national comparative risk assessment of 79 behavioural, environmental and occupational, and metabolic risks or clusters of risks in 188 countries, 1990–2013: a systematic analysis for the Global Burden of Disease Study 2013. Lancet. 2015;386(10010):2287‐2323. 26364544 10.1016/S0140-6736(15)00128-2 PMC4685753

[18] Quality and Outcomes Framework . NHS Digital. 2018. https://qof.digital.nhs.uk/search/index.asp. Accessed 08.01.2019.

[19] National Clinical Guideline Centre (UK) . Hypertension: the Clinical Management of Primary Hypertension in Adults. London, UK: Royal College of Physicians; 2011.

[20] NICE . Hypertension in adults: diagnosis and management. 2019. Accessed 08.02.2021.

[21] Perkovic V , Rodgers A . Redefining blood‐pressure targets — SPRINT starts the marathon. N Engl J Med. 2015;373(22):2175‐2178. 26551394 10.1056/NEJMe1513301

[22] Joffres M , Falaschetti E , Gillespie C , et al. Hypertension prevalence, awareness, treatment and control in national surveys from England, the USA and Canada, and correlation with stroke and ischaemic heart disease mortality: a cross‐sectional study. BMJ Open. 2013;3(8):e003423. 10.1136/bmjopen-2013-003423 PMC3758966 23996822

[23] Hart JT . Rule of halves: implications of increasing diagnosis and reducing dropout for future workload and prescribing costs in primary care. Br J Gen Pract. 1992;42(356):116‐119. 1493028 PMC1371996

[24] Psaty BM , Smith NL , Siscovick DS , et al. Health outcomes associated with antihypertensive therapies used as first‐line agents: a systematic review and meta‐analysis. JAMA. 1997;277(9):739‐745. 9042847

[25] Blood Pressure Lowering Treatment Trialists C , Sundstrom J , Arima H , et al. Blood pressure‐lowering treatment based on cardiovascular risk: a meta‐analysis of individual patient data. Lancet 2014;384(9943):591‐598. 25131978 10.1016/S0140-6736(14)61212-5

[26] Patton M . Qualitative Evaluation and Research Methods. UK: Sage Publications; 1990.

[27] Glaser B . The constant comparative method of qualitative analysis. Soc Probl. 1965;12(4):436‐445.

[28] Malterud K , Siersma VD , Guassora AD . Sample size in qualitative interview studies: guided by information power. Qual Health Res. 2015;26(13):1753‐1760. 10.1177/1049732315617444 26613970

[29] McCambridge J , Witton J , Elbourne DR . Systematic review of the Hawthorne effect: new concepts are needed to study research participation effects. J Clin Epidemiol. 2014;67(3):267‐277. 24275499 10.1016/j.jclinepi.2013.08.015 PMC3969247

[30] Constitution TNHS , Lea W . https://www.gov.uk/government/uploads/system/uploads/attachment_data/file/448466/NHS_Constitution_WEB.pdf. Published 2015. Updated 24.08.2015. Accessed.

[31] Shared Decision Making. BMJ Group. http://sdm.rightcare.nhs.uk/. Accessed.

[32] Johnson RA , Huntley A , Hughes RA , et al. Interventions to support shared decision making for hypertension: a systematic review of controlled studies. Health Expect. 2018;21(6):1191‐1207. 30221454 10.1111/hex.12826 PMC6250885

[33] Rothberg MB , Sivalingam SK , Kleppel R , Schweiger M , Hu B , Sepucha KR . Informed decision making for percutaneous coronary intervention for stable coronary disease. JAMA Intern Med. 2015;175(7):1199‐1206. 25984988 10.1001/jamainternmed.2015.1657

[34] Thompson AGH . The meaning of patient involvement and participation in health care consultations: a taxonomy. Soc Sci Med. 2007;64(6):1297‐1310. 17174016 10.1016/j.socscimed.2006.11.002

[35] Mahmoodi N , Sargeant S . Shared decision‐making ‐ Rhetoric and reality: Women's experiences and perceptions of adjuvant treatment decision‐making for breast cancer. J Health Psychol. 2019;24(8):1082‐1092. 28810390 10.1177/1359105316689141

[36] Gattellari M , Voigt KJ , Butow PN , Tattersall MH . When the treatment goal is not cure: are cancer patients equipped to make informed decisions? J Clin Oncol. 2002;20(2):503‐513. 11786580 10.1200/JCO.2002.20.2.503

[37] Elwyn G , Edwards A , Wensing M , Hibbs R , Wilkinson C , Grol R . Shared decision making observed in clinical practice: visual displays of communication sequence and patterns. J Eval Clin Pract. 2001;7(2):211‐221. 11489045 10.1046/j.1365-2753.2001.00286.x

[38] Frosch DL , May SG , Rendle KA , Tietbohl C , Elwyn G . Authoritarian physicians and patients' fear of being labeled 'difficult' among key obstacles to shared decision making. Health Aff (Millwood). 2012;31(5):1030‐1038. 22566443 10.1377/hlthaff.2011.0576

[39] Jansen J , McKinn S , Bonner C , Muscat DM , Doust J , McCaffery K . Shared decision‐making about cardiovascular disease medication in older people: a qualitative study of patient experiences in general practice. BMJ Open. 2019;9(3):e026342. 10.1136/bmjopen-2018-026342 PMC6475217 30898831

[40] High blood pressure (hypertension). NHS England http://onlinelibrary.wiley.com/doi/10.1002/14651858.CD001431.pub5/full. Accessed. 25.09.2017, 2017.

[41] Treadwell JS , Wong G , Milburn‐Curtis C , Feakins B , Greenhalgh T . GPs’ understanding of the benefits and harms of treatments for long‐term conditions: an online survey. BJGP Open. 2020;4(1):bjgpopen20X101016. 10.3399/bjgpopen20X101016 PMC7330197 32127362

[42] Denness C . What are consultation models for? InnovAiT: Educ Inspir Gen Pract. 2013;6(9):592‐599.

[43] Harper C , Ajao A . Pendleton’s consultation model: assessing a patient. Br J Community Nurs. 2010;15(1):38‐43. 20216519 10.12968/bjcn.2010.15.Sup5.78117

[44] Kurtz S , Silverman J , Benson J , Draper J . Marrying content and process in clinical method teaching: enhancing the Calgary‐Cambridge guides. Acad Med. 2003;78(9):802‐809. 12915371 10.1097/00001888-200308000-00011

